# Primary Bone Marrow Classic Hodgkin Lymphoma With Haemophagocytic Lymphohistiocytosis

**DOI:** 10.1002/jha2.70014

**Published:** 2025-06-19

**Authors:** Chun‐Tsu Lee

**Affiliations:** ^1^ Department of Haematology‐Oncology National University Cancer Institute Singapore Singapore; ^2^ Department of Medicine Yong Loo Lin School of Medicine National University Singapore Singapore Singapore; ^3^ Department of Medicine Alexandra Hospital, National University Health System Singapore Singapore

1

A previously well, 25‐year‐old man was referred by his GP for persistent fever, anorexia, night sweats and weight loss for the past 1 month. He was febrile and lethargic, with no palpable hepatosplenomegaly or lymphadenopathy. An initial CT scan showed no suspicious masses or organomegaly. Full blood count showed pancytopenia with a haemoglobin of 8.8 g/dL, platelet count of 50 × 10^9^/L and leucocyte count of 1.5 × 10^9^/L (neutrophil 0.8 × 10^9^/L). Liver function tests were deranged with raised bilirubin and transaminases. Marked hyperferritinemia of 20,000 ug/L and high LDH of 1080 IU/mL were observed together with prolonged PT and APTT with hypofibrinogenemia of 1.1 g/L and hypertriglyceridemia of 6.8 mmol/L. Autoimmune workup, plasma HIV and EBV viral load were negative. Bone marrow (BM) aspirate was performed, showing the presence of Reed–Sternberg cells and Hodgkin cells, characteristics of classical Hodgkin Lymphoma (HL). There were increased histiocytic activities with brisk hemophagocytic activities of note (Figure 1). Histology showed lymphomatous infiltration featuring scattered atypical medium‐sized to large cells with round to lobated nuclei and occasional prominent nucleoli, accompanied by small lymphocytes, occasional plasma cells, eosinophils and neutrophils (Figure 1). Immunohistochemistry showed scattered large CD30+ neoplastic cells that co‐express PAX5 but not CD20, CD2 nor CD3, accompanied by numerous CD2+ CD3+ reactive T‐cells. In situ hybridization for EBER was negative. T‐cell receptor gene rearrangements showed polyclonal expansion of T‐cells. PET scan showed diffuse intense, FDG‐avid uptake was seen along the axial and proximal appendicular skeleton (SUVmax 14.1). Diffuse FDG‐avid uptake was seen involving the liver and spleen (SUVmax 5.1 vs. liver SUVmax 4.1), but they were not enlarged. No other suspicious FDG‐avid lesions or adenopathy were detected. Overall findings were compatible with primary BM HL with Haemophagocytic Lymphohistiocytosis (HLH). His condition deteriorated with worsening cytopenias and liver functions. Dexamethasone, Etoposide and IVIG were swiftly commenced to control cytokine storm and reactive T‐cell proliferation. With other supportive measures including blood products, antimicrobials and myeloid growth factors, his condition improved and he was started on definitive chemotherapy consisting of Brentuximab vedotin plus Doxorubicin, Vinblastine and Dacarbazine. He achieved a complete metabolic response after six cycles of chemotherapy.

**FIGURE 1:**
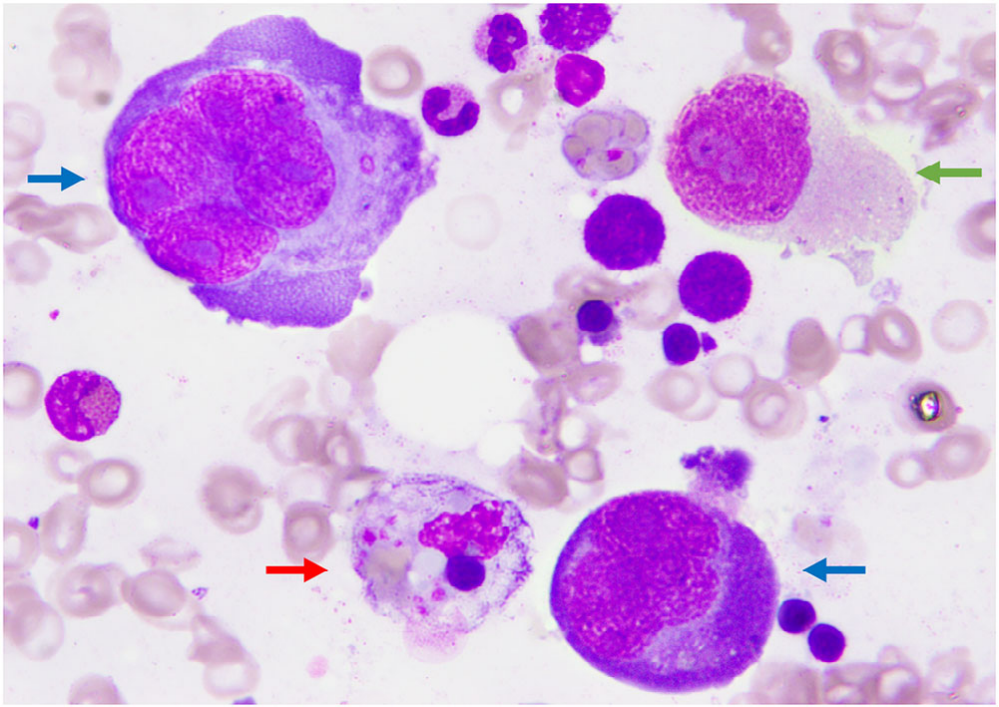
Bone marrow aspirate showed a rare finding of giant, multinucleated Reed Sternberg cells with fine chromatin structure and prominent four nucleoli. They have basophilic cytoplasm with perinuclear clearing (indicated in blue arrows). In addition, there are mononuclear variants of Reed–Sternberg cells, called the Hodgkin cells (indicated by green arrow). They have agranular cytoplasm with round and elongated nuclei and prominent nucleoli. Histiocytic activities were increased with brisk haemophagocytic activities of note. A macrophage with phagocytosed platelets, mature and nucleated erythrocytes was observed adjacent to the neoplastic cells (indicated in red arrow). (Wright‐Giemsa stain, 100× oil immersion objective.)

In the era of PET scanning, BM biopsies are rarely conducted for HL. Nevertheless, it is unusual to detect Reed–Sternberg and Hodgkin cells in an aspirate because the lymphomatous infiltration induces marrow fibrosis, hampering the aspiration process. BM involvement by HL is observed in 5%–15% of patients and is usually associated with lymphadenopathy [1, 2]. Primary BM HL is rare (0.25%) and usually occurs in HIV‐positive patients and is EBV‐related [2]. The occurrence in HIV‐ and EBV‐negative patients was exceedingly rare [1]. The prognosis of patients with secondary BM involvement is not worse than the prognosis of other advanced HL patients [2]. But the prognosis of isolated BM involvement in HL is not well established. Secondary HLH from HL was reported previously to be approximately 6%–8.9% [3, 4]. Patients with secondary HLH from HL have worse prognosis and higher mortality compared to patients without HLH due to delayed diagnosis [4]. Early recognition and control of cytokine storm and organ failure is vital prior definitive chemoimmunotherapy for HL [3].

## Author Contributions

C.T.L. contributed to the patient's diagnosis, care, data interpretation and the writing of the manuscript.

## Ethics Statement

The patient has consented and signed the patient consent permission form.

## Consent

Patient's consent has been obtained for the publication of patient's clinical history, diagnostic laboratory and radiologic results.

## Conflicts of Interest

The author declares no competing interests.

## Data Availability

The data that support the findings of this study are available from the corresponding author upon reasonable request.

## References

[1] C. Laurent , D. A. Arber , P. Johnston , F. Fend , A. Zamo , and A. D. Attygalle , “Diagnosis of Classic Hodgkin Lymphoma on Bone Marrow Biopsy,” Histopathology 76, no. 7 (2020): 934–941, 10.1111/his.14085. Epub 2020 May 7. 32092168

[2] R. Munker , D. Hasenclever , O. Brosteanu , E. Hiller , and V. Diehl , “Bone Marrow Involvement in Hodgkin's Disease: An Analysis of 135 Consecutive Cases. German Hodgkin's Lymphoma Study Group,” Journal of Clinical Oncology 13, no. 2 (1995): 403–409, 10.1200/JCO.1995.13.2.403. 7844601

[3] J. Knauft , T. Schenk , T. Ernst , et al., “Lymphoma‐associated Hemophagocytic Lymphohistiocytosis (LA‐HLH): A Scoping Review Unveils Clinical and Diagnostic Patterns of a Lymphoma Subgroup With Poor Prognosis,” Leukemia 38 (2024): 235–249, 10.1038/s41375-024-02135-8. 38238443 PMC10844097

[4] Z. Jin , Y. Wang , N. Wei , and Z. Wang , “Hodgkin Lymphoma‐Associated Hemophagocytic Lymphohistiocytosis—A Dangerous Disease,” Annal of Hematology 99, no. 7 (2020): 1575–1581, 10.1007/s00277-020-04093-4. 32500223

